# The German Version of the Gaze Anxiety Rating Scale (GARS): Reliability and Validity

**DOI:** 10.1371/journal.pone.0150807

**Published:** 2016-03-03

**Authors:** Gregor Domes, Lisa Marx, Ines Spenthof, Markus Heinrichs

**Affiliations:** Department of Psychology, Laboratory for Biological and Personality Psychology, University of Freiburg, Freiburg, Germany; Eberhard Karls University of Tuebingen Medical School, GERMANY

## Abstract

**Objective:**

Fear of eye gaze and avoidance of eye contact are core features of social anxiety disorders (SAD). To measure self-reported fear and avoidance of eye gaze, the Gaze Anxiety Rating Scale (GARS) has been developed and validated in recent years in its English version. The main objectives of the present study were to psychometrically evaluate the German translation of the GARS concerning its reliability, factorial structure, and validity.

**Methods:**

Three samples of participants were enrolled in the study. (1) A non-patient sample (n = 353) completed the GARS and a set of trait questionnaires to assess internal consistency, test-retest reliability, factorial structure, and concurrent and divergent validity. (2) A sample of patients with SAD (n = 33) was compared to a healthy control group (n = 30) regarding their scores on the GARS and the trait measures.

**Results:**

The German GARS fear and avoidance scales exhibited excellent internal consistency and high stability over 2 and 4 months, as did the original version. The English version’s factorial structure was replicated, yielding two categories of situations: (1) everyday situations and (2) situations involving high evaluative threat. GARS fear and avoidance displayed convergent validity with trait measures of social anxiety and were markedly higher in patients with GSAD than in healthy controls. Fear and avoidance of eye contact in situations involving high levels of evaluative threat related more closely to social anxiety than to gaze anxiety in everyday situations.

**Conclusions:**

The German version of the GARS has demonstrated reliability and validity similar to the original version, and is thus well suited to capture fear and avoidance of eye contact in different social situations as a valid self-report measure of social anxiety and related disorders in the social domain for use in both clinical practice and research.

## Introduction

The face, especially the eye region, provides a multitude of social information [1]. Eye contact is an important non-verbal signal in social interactions, e.g. it influences the degree of perceived intimacy and dominance, and it influences the perception of a person’s social competence [2]. Direct eye-contact is known to facilitate emotion recognition from facial expressions [3].

Fear and avoidance of eye-to-eye contact is a frequently reported clinical symptom of Social Anxiety Disorder (SAD). One explanation for reduced eye gaze in SAD relates to the need to avoid a possible source of threat, as the eyes convey socially evaluative information [4]. From an evolutionary perspective, gaze avoidance in SAD could be an adaptive behavior to avoid competition or social threat related to expected scrutiny [5–7]. Since individuals with SAD tend to perceive themselves as inferior, they are less motivated to compete with others, and are more likely to appease their counterpart by avoiding direct eye-contact, thereby expressing submissiveness [5,7,8].

In using systematic observer ratings, some studies indeed reported inadequate eye contact in SAD compared to healthy controls [9–11]. The largest effects were found under conditions involving social evaluative threat and interactions with the opposite sex [10]. Another set of studies used eye tracking to measure gaze behavior in SAD and reported reduced fixations on the faces’ eye region, while the strongest effects were detected for faces expressing negative emotions [4,12,13]. Symptom severity in SAD is associated with gaze avoidance [4,13,14], and gaze avoidance is associated with self-reported negative affect [8]. However, several other studies could not replicate these findings [15,16].

Despite this growing evidence for reduced eye gaze in SAD relying on objective experimental data, less is known about the subjective experience of eye contact in SAD. Schneier et al. [17] recently published the Gaze Aversion Rating Scale (GARS) as a self-report measure of fear and avoidance of eye gaze. Preliminary results demonstrate good reliability of the GARS and support the hypothesis that patients with generalized social anxiety disorder (GSAD) report significantly higher levels of fear and avoidance of eye gaze in various social situations than matched healthy controls. A follow-up study employing a factor analysis of GARS ratings distinguished two categories of social situations assessed with the GARS: (1) everyday social situations and (2) those characterized by social threat or interactions with dominant others [18].

We aimed in this study to assess the psychometric properties of the German translation of the GARS (its reliability, convergent and discriminant validity), to replicate the factorial structure of the GARS, and to further assess self-reported fear and avoidance of eye gaze in a sample of socially anxious individuals and matched healthy controls.

## Methods

### Participants

The first sample of participants for evaluating reliability and factorial structure of the GARS comprised n = 353 adults recruited via local on-campus advertising. This sample mainly comprised students (n = 234; 66.3%), and employees from different professions (n = 89; 27.1%) aged a mean 29.4 years (+/- s.d. = 10.9; min-max = 18–68 years). About two-thirds of the sample were women (n = 216; 61.2%) and most had higher education (n = 301; 85.3%). Psychology students were excluded, as were participants reporting a lifetime history of mental illness or current severe somatic illness. To determine the test-retest reliability, two non-overlapping subsamples of this group joined the second enrollment 2 months (n = 76) and 4 months (n = 73) after the first survey.

In all, 1208 persons were contacted by e-mail, of whom 436 responded by starting the survey. Of these, 359 completed the first evaluation questionnaire yielding 353 valid data sets for reliability analysis, and 328 data sets for correlational analyses. From the initial sample, a random subsample of 156 persons was contacted by e-mail at the first retest interval (2 months), 105 responded, and 76 completed the second survey. For the second retest interval (4 months), we contacted another subsample of 156 persons via e-mail: 90 responded, 73 completed the second survey.

To validate the GARS, we enrolled a group of n = 33 male participants with GSAD from an ongoing study unrelated to the present evaluation. The GSAD diagnosis was confirmed via the Structured Clinical Interview for DSM-IV (SKID [19]). GSAD patients had no current MDD, substance abuse, other anxiety disorder, or lifetime psychotic or bipolar disorder, and were not taking psychoactive medication. An age-matched healthy control group of n = 30 men was recruited via a local newspaper advertisement. The control group presented no current diagnosis of an axis-I or axis-II disorder according to the DSM-IV and confirmed by clinical interviews using the SCID.

### Ethics statement

The procedures in this study were approved by the local ethics committee of the University of Freiburg. All participants gave written informed consent before participation and received monetary compensation.

### Procedures

All participants completed the GARS, and a battery of questionnaires comprising questionnaires on demographic variables, trait anxiety, social anxiety, and personality traits within approx. 30 minutes.

The GARS was originally developed from unstructured interviews with GSAD patients and comprises 17 situations involving enhanced fear or avoidance of eye contact reported by these patients [17]. For each of these situations, fear and avoidance are rated on a 4-point Likert scale (0 = none to 3 = severe). These ratings are summed up to subscores of GARS-fear and GARS-avoidance and an overall GARS-total score. Additional items on associated cognitions and the course of gaze anxiety are included but were not subject to the original evaluation studies [17,18]. The original version’s reliability is reported to be very high, with Cronbach’s α ranging from .88 to .96. Eight-week test-retest reliability was estimated for a small subsample of healthy controls at r_12_ = .99 [17]. To draft the German version, we had the original questionnaire translated by a professional translator and then back translated by an independent English native-speaker. Any divergence between the initial version and back-translated version were resolved in an expert panel discussion until consensus was reached about how to modify the German version for conceptual equivalence to the English version. The final version (see S1 Appendix) was then prescreened by n = 15 undergraduate students for understandability and clarity.

In addition to the GARS, participants completed the Social Interaction Anxiety Scale (SIAS [20]), a widely used dimensional measure of social anxiety. The 20-item self-report questionnaire has good to excellent reliability and validity. The German version employed in this study has shown psychometric properties similar to the original version’s [21].

The Brief Fear of Negative Evaluation—revised (BFNE-R) is a 12-item self-report questionnaire measuring fear and distress related to negative evaluation by others on 4-point Likert scales The revised brief version is based on the original Fear of Negative Evaluation Scale [22] and has demonstrated good reliability and validity [23]. The translated German version employed in the present study is being published [24] and has revealed similarly good reliability and validity as the original version.

General trait anxiety was assessed via the trait scale of the State-Trait Anxiety Inventory (STAI-t [25]). This well-known measure of anxiety has been extensively used and validated in numerous studies. It consists of 20 statements rated on 4-point Likert scales. Good to excellent reliability and validity have been reported for the German version [26].

Depressive symptoms were assessed with the German version of the Center of Epidemiological Studies Depression Scale (CES-D [27]). The short German version employed in the present study measures the depressive symptom burden on 15 items with 4-point Likert scales, and has displayed good reliability and validity for measuring the severity of depressive symptoms in the general population [28].

Finally, a 30-item version of the Neo-Five-Factor-Inventory (NEOFFI [29]) was employed to assess broad personality dimensions (“Big Five”): Extraversion, Neuroticism, Openness, Conscientiousness, and Agreeableness. The 30-item version comprises a subset of the original items and possesses psychometric properties similar to the long version’s [30].

A subsample of the initial group was re-assessed via the GARS two months after the first assessment, and another independent subsample completed the GARS four months a second time. All questionnaires were presented with EFS survey 10.6 using secure connection via the internet (Questback, Cologne, Germany). Each participant was sent a single, unique keycode by e-mail to access the online survey and complete the questionnaires at home.

### Analysis

Reliability was estimated by calculating internal consistencies (Cronbach’s α) for the subscales and total score of the GARS. In addition, item-total correlations were calculated and split-half-reliabilities using the odd-even-methods with Spearman-Brown correction. Test-retest reliabilities were estimated by calculating intraclass correlation coefficient (ICC) and Pearson-correlations for two retest intervals: at 2 and 4 months.

The factorial structure was determined by calculating two independent principal component analysis (PCA) with a Varimax rotation for GARS-fear and GARS-avoidance ratings separately. A confirmatory approach with a two-factor solution was chosen to replicate the preliminary findings with the original English version [17,18].

Convergent and discriminant validity was investigated by calculating the bivariate correlation coefficient of the GARS subscales with the SIAS, BFNE, STAI-T, CES-D and the subscales of the NEOFFI-30. We also explored the predictive power of the two factors using correlation analyses.

Finally, we tested criterion-related validity by comparing the GARS subscales in a group of participants with SAD to a healthy control group. For this purpose, *t*-tests were calculated for the fear and avoidance subscales, the total score and the two factors revealed in the PCA.

All analyses were conducted with SPSS for Windows (Version 22). Significance threshold was *p<*.*05*. Correction for multiple testing was achieved by correcting the exact p-values according to Bonferroni.

## Results

### Descriptive Statistics

Descriptive data of the item-analysis are illustrated in Table 1. As expected, some situations were rated higher than others with regard to the perceived fear and avoidance of eye contact. Item-total correlations were in the medium range between r = .32 and r = .70.

**Table 1:** Item analysis. Mean (+/- s.d.) values and item-total correlations (r_it_).

		Fear			Avoidance		
No.	Item	mean	s.d.	r_it_	mean	s.d.	r_it_
1.	Giving a speech	1.01	0.84	.53	0.99	0.86	.53
2.	Speaking to a group of people at a party	0.58	0.75	.60	0.53	0.72	.62
3.	Speaking up at a meeting	0.95	0.88	.64	0.84	0.83	.63
4.	Speaking in a discussion with a few people	0.69	0.82	.64	0.62	0.78	.64
5.	Dealing with a cashier	0.17	0.50	.48	0.25	0.59	.43
6.	Being introduced	0.39	0.68	.64	0.29	0.56	.57
7.	Greeting an acquaintance passing by on the street	0.28	0.63	.52	0.30	0.60	.43
8.	Speaking with someone you don't know well	0.69	0.76	.67	0.65	0.73	.62
9.	Speaking to someone you find attractive	1.15	0.96	.70	1.00	0.92	.64
10.	Inviting someone you don’t know well…	0.98	0.90	.68	0.85	0.82	.70
11.	Feeling close to someone you love	0.17	0.47	.38	0.20	0.48	.35
12.	Discussing the quality of your work with …	1.04	0.90	.66	0.92	0.84	.59
13.	Having a routine talk with a close family member	0.13	0.42	.47	0.20	0.61	.39
14.	Listening while a person speaks to you, in general	0.22	0.52	.56	0.36	0.61	.49
15.	Speaking while a person listens to you, in general	0.43	0.68	.67	0.56	0.76	.63
16.	Expressing a disagreement	0.84	0.86	.57	0.81	0.85	.56
17.	Receiving a compliment	0.91	0.93	.63	1.11	0.95	.56

Calculation of GARS-fear and GARS-avoidance sub-scores resulted in comparable mean values (mean+/- s.d.: GARS-fear: 10.6 +/- 8.3; GARS-avoidance: 10.5 +/- 10.5; GARS-total: 21.1 +/- 15.3). The correlation between GARS-fear and GARS-avoidance sub-scores was r = .78 (p<.001). Age was not associated with the GARS-fear score (r = -.03; p = .59), but there was a small yet significant negative correlation for the GARS-avoidance (r = -.16; p = .002). Educational level was not associated with the GARS scales (all p>.50). Women revealed slightly higher scores on both subscales and the total score (GARS-fear: men: 9.4 +/- 8.4, women: 11.4 +/- 8.2, t[351] = 2.27; p = .024; GARS-avoidance: men: 9.3 +/- 7.6, women: 11.2 +/- 7.6, t[351] = 2.28; p = .023; GARS-total: men: 18.7 +/- 14.7, women: 22.6 +/- 15.3, t[351] = 2.41; p = .016).

### Reliability

Internal consistencies (Cronbach’s α) for the subscales and the total score were very high, ranging from .90 to .95. Accordingly, split-half reliabilities of the subscores and total scores was very high, ranging from r = .82 to r = .86 for uncorrected, and from r = .91 to r = .93 for Spearman-Brown corrected correlations. Finally, test-retest correlations over an interval of 2 and 4 months revealed high to medium stability of the test scores in two independent samples (see Table 2).

**Table 2:** Reliability of the GARS. Internal consistency, split-half reliability and test-retest-reliability of the subscales and the total score.

					Test-Retest-Reliability			
			Split-Half Reliability		Pearson Corr.		ICC^b^	
	Cronbach’s α (n = 353)	r_it_ range (n = 353)	uncorr. (n = 353)	corr.^a^ (n = 353)	2-months	4-months	2-months (n = 76)	4-months (n = 73)
GARS-Fear	.91	.38–.70	.86	.93	.86	.73	.86 (.79–.91)	.72 (.58–.81)
GARS-Avoidance	.90	.35–.70	.83	.93	.79	.71	.76 (.64–.84)	.71 (.58–81)
GARS-Total	.95	.32–.70	.82	.91	.87	.72	.86 (.78–.91)	.70 (.56–.80)

Note.

^a^ Spearman-Brown correction;

^b^ Intraclass correlation coefficient (ICC), 95% CI is given in brackets.

### Factorial structure

The confirmatory factor analysis for the GARS-fear ratings with Varimax rotation revealed two distinct factors with high loadings (r>.30) of 14 items. Three items showed substantial loadings on both factors. Explained variance of the rotated solution was 52.8 percent. In the GARS-avoidance ratings, 13 of the 17 items showed distinct loadings (r>.30) on one of the two factors. The two-factor solution explained 46.4 percent of the variance observed (factor loadings are given in Table 3).

**Table 3:** Factorial structure of the GARS. Factor loadings of the items in the two separate 2-factor solution confirmatory PCA with Varimax rotation for GARS-Fear and GARS-Avoidance. Only loadings with r>.30 are given.

		GARS-Fear		GARS-Avoidance	
	Item	Factor 1	Factor 2	Factor 1	Factor 2
5.	Dealing with a cashier	.744		.690	
6.	Being introduced	.655		.517	.420
7.	Greeting an acquaintance passing by on the street	.701		.518	
11.	Feeling close to someone you love	.638		.502	
13.	Having a routine talk with a close family member	.701		.689	
14.	Listening while a person speaks to you, in general	.664		.624	
1.	Giving a speech		.722		.607
3.	Speaking up at a meeting		.741		.725
4.	Speaking in a discussion with a few people		.635		.674
9.	Speaking to someone you find attractive		.694		.668
10.	Inviting someone you don’t know well…		.744		.678
12.	Discussing the quality of your work with a boss or a teacher		.692		.684
16.	Expressing a disagreement		.714		.725
17.	Receiving a compliment		.652		.584
2.	Speaking to a group of people at a party	.496	.452	.408	.551
8.	Speaking with someone you don't know well	.401	.598	.418	.547
15.	Speaking while a person listens to you, in general	.507	.534	.471	.512

The factors revealed by the two factor analyses capture fear and avoidance of eye contact in two situational categories: (1) everyday situations (e.g. “dealing with a cashier”, “having a routine talk with a close family member”) with 6 items, (2) situations involving high levels social threat (e.g. “speaking up at a meeting”, “inviting someone you don’t know well…”) with 8 items. Subscores calculated by summing up the items on each factor correlated positively for the fear ratings (r = .56; p<.001) and avoidance ratings (r = .53; p<.001).

Exploring sex differences in fear of eye gaze in the different situational factors using a 2-way ANOVA, we noted a significant sex-by-situation interaction (F[1,351] = 23.84; p<.001). Post-hoc tests revealed significantly higher levels of fear in women in conjunction with social-threat situations (t[351] = 3.44; p<.001), but not in everyday situations (t[351] = 0.75; p = .46). We observed the same pattern in the avoidance ratings (sex-by-situation interaction: F[1,351] = 18.18; p<.001).

### Validity—Convergent and discriminant validity

Correlation analyses revealed positive correlations with trait anxiety, depression, fear of negative evaluation, and social anxiety for both the fear and avoidance sub-scores of the GARS. Unsurprisingly, we observed the highest correlation for social anxiety, confirming the diagnostic relevance of reduced eye contact in social phobia (see Table 4). Divergent validity was confirmed by the negative correlation with extraversion, conscientiousness and to some extent agreeableness. However, negative correlations were much lower, suggesting that normal variants in these variables are less predictive for fear-associated reductions in eye contact.

**Table 4:** Concurrent and discriminant validity of the GARS. Correlations of the total scale (Tot), factor 1 (F1 “Everyday situations”) and factor 2 (F2 “Situations with social threat”) with social anxiety (SIAS), fear of negative evaluation (BFNE), trait anxiety (STAI-t), depression (CES-D), and the big five personality dimensions (n = 328).

	GARS-fear			GARS-avoidance		
Scale	Tot	F1	F2	Tot	F1	F2
SIAS	.63	.42	.63	.64	.43	.61
BFNE	.36	.20	.38	.34	.21	.34
STAI-t	.45	.31	.44	.42	.31	.39
CES-D	.37	.26	.36	.36	.30	.33
Neuroticism	.43	.27	.42	.43	.31	.40
Extraversion	-.32	-.24	-.29	-.30	-.22	-.28
Conscientiousness	-.25	-.19	-.22	-.20	-.16	-.18
Openness	-.03	-.02	-.03	-.09	-.03	-.09
Agreeableness	-.09	-.13	-.05	-.15	-.21	.11

The association between the total GARS score and social anxiety (SIAS) remained significant after controlling for trait anxiety (STAI-t): GARS-total/SIAS r_part_ = .56. Using the situational factors for correlation analyses, we found higher correlations for situations of social threat than everyday situations. In particular, fear and avoidance of eye gaze in socially-threatening situations (factor 2) were the better predictors for social anxiety (r = .63 and r = .61) than fear and avoidance in everyday situations (factor 1; r = .42 and r = .43; Fisher’s Z = 3.74; p<.001).

### Validity—Gaze anxiety and avoidance in GSAD

**Fig 1:**
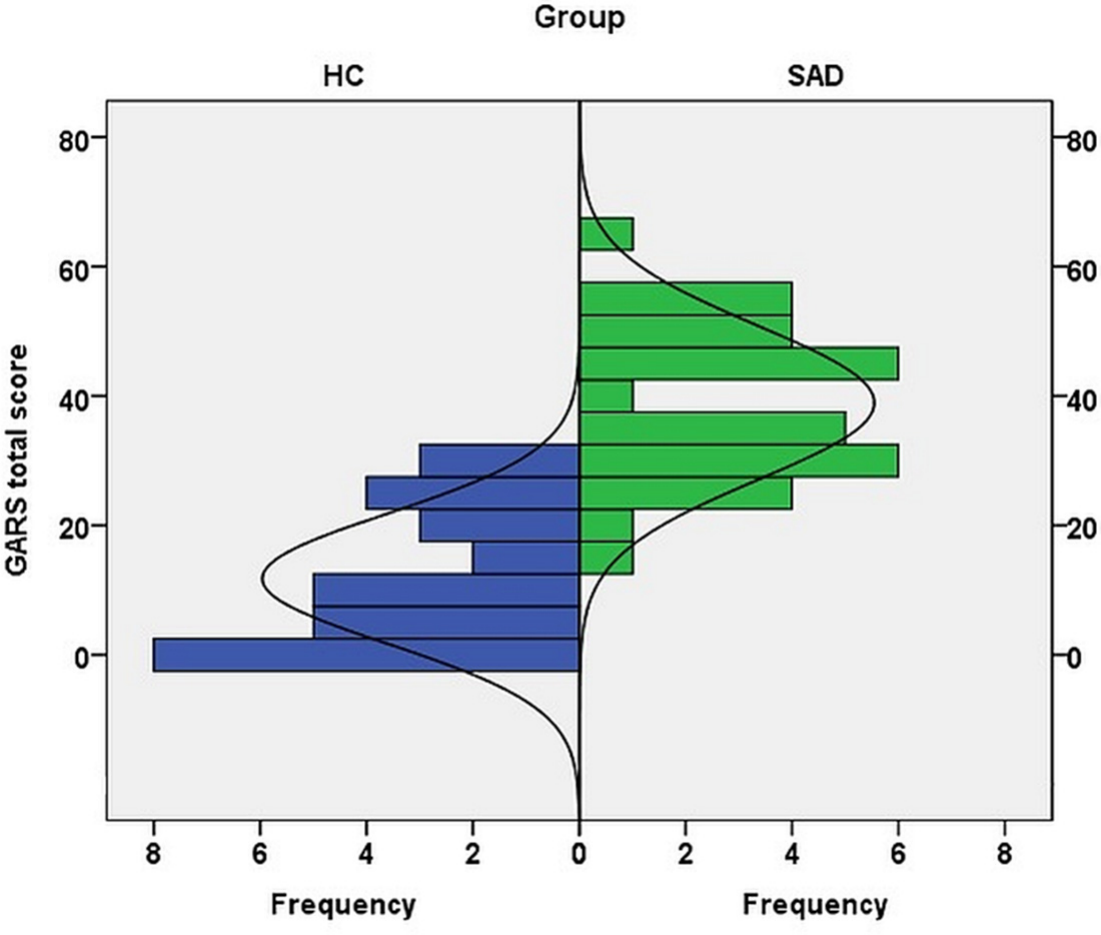
Distribution of GARS scores in a sample of SAD patients (n = 33) and matched healthy controls (n = 30).

Group differences between GASD patients and matched healthy controls were calculated for the GARS subscales and the two situational factors (see Table 5). Overall, GSAD patients reported higher levels of gaze fear and avoidance than healthy controls (Fig 1). Effect size (Cohen’s d) was 2.47, indicating an overlap of the two distributions of about 23%.

**Table 5:** Fear and avoidance of eye gaze in GSAD and matched healthy controls.

	GSAD (n = 33)		HC (n = 30)		Statistics		
Scale	Mean	s.d.	mean	s.d.	t	p	d
GARS Fear	19.6	6.4	5.2	5.5	9.57	<.001	2.41
GARS Avoidance	19.3	6.3	6.6	5.9	8.21	<.001	2.08
GARS Total	38.9	11.9	11.8	10.0	9.76	<.001	2.47
GARS Fear							
Factor 1	0.59	0.43	0.12	0.20	5.53	<.001	1.06
Factor 2	1.54	0.45	0.45	0.46	9.53	<.001	2.40
GARS Avoid.							
Factor 1	0.65	0.42	0.16	0.27	5.38	<.001	1.39
Factor 2	1.49	0.46	0.56	0.47	7.91	<.001	2.00
Age	28.7	9.5	27.0	6.1	0.83	n.s.	0.21
STAI (20–80)	49.7	9.9	33.9	8.9	6.60	<.001	1.68
SIAS-D (0–68)	32.3	10.7	6.8	7.4	10.26	<.001	2.77
BFNE (12–60)	44.6	7.3	27.5	9.4	8.14	<.001	2.03
BDI-II (0–60)	2.7	2.5	0.5	1.0	4.46	<.001	1.16
Neuroticism	14.3	4.6	5.8	3.9	7.83	<.001	1.99
Extraversion	10.8	3.6	15.7	3.7	-5.21	<.001	-1.34
Conscientiousness	15.5	3.7	18.8	3.5	-3.55	.002	-0.92
Openness	15.8	4.5	15.7	5.1	0.07	n.s.	0.02
Agreeableness	15.0	3.6	15.1	4.3	-0.04	n.s.	-0.03

As expected, the GSAD patients displayed markedly enhanced fear and avoidance in both everyday (factor 1) and social threat situations (factor 2). However, a three-way ANOVA with group (HC vs. GSAD), scale (fear vs. avoidance), and situation (everyday vs. threat) revealed a significant group x situation interaction (F[1,61] = 27.68; p<.001), indicating a stronger tendency to report high levels of fear and avoidance of eye gaze in threatening situations in GSAD, as compared to healthy controls. Calculation of effect sizes (Cohen’s d) confirmed the greater difference in gaze fear and avoidance in GSAD patients in social threat situations (factor 2).

Exploratory analysis of the descriptive data in the additional GARS items revealed consistently higher scores on the anxiety-associated attributions of gaze avoidance in patients with GSAD (see Table 6).

**Table 6:** Comparison of GSAD patients and matched healthy controls on the additional GARS items.

		GSAD (n = 33)		HC (n = 30)		Statistics		
	Item	mean	s.d.	mean	s.d.	t	p	d
18.	I avoid eye contact because it make me anxious	1.09	1.01	0.27	0.45	4.11	<.001	1.05
19.	I avoid eye contact only because it interferes with my concentration	1.00	0.97	0.77	0.90	0.99	n.s.	0.25
20.	I feel self-conscious when I make eye contact.	1.64	0.86	0.37	0.62	6.68	<.001	1.69
21.	I am concerned that I stare too much into others' eyes.	1.73	1.07	0.93	0.94	3.11	.003	0.79
22.	I have difficulty deciding how much eye contact is best.	2.27	0.88	0.90	1.00	5.82	<.001	1.45
23.	Making eye contact is important for my social and work relationships	1.70	0.95	2.27	0.94	-2.38	.020	-0.60

Interestingly, GSAD were less convinced that making eye contact is important for their social interactions (item 23). Participants with GSAD reported a large range (4 to 45 years) concerning the age of onset of their problems making eye contact (item 24 “Earliest age when I felt anxious about eye contact or avoiding eye contact”), but most reported that they started having difficulty before their 18^th^ birthday (77%). Regarding how their difficulties developed from childhood to adulthood (item 25: “My current anxiety and avoidance related to eye contact is… (0) worse than in my childhood … (3) a lot better than in my childhood”), 39% of the GSAD patients reported worsened, 26% unchanged, and 35% less gaze anxiety.

## Discussion

The present study investigated the psychometric properties of the German version of the GARS in a sample of non-patients and in patients with GSAD. Our data provides evidence of excellent internal consistency and high reliability over several months. The factorial structure of the original English version was replicated with the German version and reveals the two distinct “everyday situations” vs. “situations with evaluative threat”-factors. We noted a strong linear association between fear and avoidance of eye contact with social anxiety in non-patients. Accordingly, fear and avoidance of eye contact was higher in patients with GSAD than in healthy controls. Overall, the psychometric properties of the German version of the GARS verify the usefulness of this self-report questionnaire to measure the subjective experience of eye contact in various situations.

Our assessment of internal consistency and test-retest reliability revealed that the German GARS is as reliable as the original version [17]. The stability of fear and avoidance of eye contact over intervals of 2 and 4 months was high and similar to results already reported [17,18]. Fear and avoidance showed a moderate to high correlation (r = .78) with about 60% shared variance. Thus, the calculation of a total score by summing up both aspects of gaze anxiety seems justified, although there is a considerable amount of unique variance in fear and avoidance. The differentiation between fear and avoidance might be especially relevant in individuals exhibiting gaze avoidance without perceiving high levels of fear of eye contact, e.g. in some patients with autism spectrum disorder.

The factorial structure of the GARS in the present study concurs overall with previous reports [18]. For both fear and avoidance of eye contact, the two-factor solutions explained about 50% of the observed variance and revealed a nearly identical loading pattern, not surprising given the correlation between fear and avoidance ratings. The factors comprised distinct groups of items, and might be interpreted as reflecting “everyday situations” (factor 1) and “situations with social threat” (factor 2) that correspond to the two factors called “GARS-general” and “GARS-dominance” by Langer et al. [18]. The two factors differed in predictive power for social anxiety measured with the SIAS: fear and avoidance of eye gaze in situations with social threat (factor 2) predicted social anxiety significantly better than fear and avoidance in everyday situations (factor 1). This stands in contrast to previous findings, as Langer et al. [18] reported the opposite pattern using the Social Phobia Scale as a criterion, a difference which might explain the inconsistency between the two studies.

As expected, GARS scores’ concurrent correlations with well-established measures of social anxiety (SIAS), general anxiety (STAI-t), fear of negative social evaluation (BFNE), and depression (CES-D) were all positive and lower to medium range. In contrast, high levels of fear and avoidance of eye contact predicted lower levels of extraversion and conscientiousness. The maximum explained variance was about 40% for social anxiety, and about 10% for extraversion, showing that self-reported fear and avoidance of eye gaze has a limited predictive power for complex personality traits. However, after controlling for general anxiety, total GARS scores still explained about 30% percent of the variance in social anxiety, suggesting a specific amount of common variance between difficulties in eye gaze and social anxiety. Notably, the association between fear of negative evaluation and fear and avoidance of eye gaze was modest, with 13% shared variance.

Women reported slightly higher levels of fear and avoidance of eye gaze in this study, a finding consistent with the higher prevalence of social anxiety in women in the general population [31]. Interestingly, the women in this study also reported higher levels of social anxiety in the SIAS and neuroticism in the NEOFFI, which is in line with previous studies [32,33]. Moreover, higher levels of fear and avoidance of eye gaze in women appear to be more pronounced in situations with evaluative threat rather than in social situations in general. In this study, we found small yet significant differences in fear and avoidance of eye contact between women and men: women reported higher levels of fear and avoidance, especially in situations involving high social-evaluative threat (factor 2). This is in line with women reporting significantly greater fear than men in situations involving high levels of social-evaluative threat, such as giving a talk in front of an audience, speaking up at a meeting, or being the center of attention in general [33].

As expected, group comparison between the GSAD patients and our age-matched control group revealed markedly higher levels of fear and avoidance in social anxiety. This effect was pronounced in conjunction with situations involving high levels of social threat (factor 2). The overall scores the GSAD group obtained in the present study were somewhat lower than those of the sample that Schneier et al. examined [17], which is most likely due to the fact that in our study only men were included, whereas the patients in the Schneier study comprised nearly equal numbers of men and women.

### Limitations

It should be noted that the GARS is based on self-reporting and thus behavioral validation; more objective measures of eye contact in social situations using appropriate techniques (e.g. eye-tracking) are still needed. Furthermore, since the present study investigated only male patients with GSAD, potential sex-specific differences in fear and avoidance of eye contact in GSAD should be specifically addressed in future studies. The specificity of gaze fear and avoidance needs to be investigated by comparing patients suffering from social anxiety with patients presenting other mental disorders involving severe social deficits, such as anxiety disorders, autism spectrum disorder, schizophrenia, or chronic depression. Finally, cultural background is a significant modulator in non-verbal communication behavior, exerting a particular influence on eye gaze behavior and the subjective response to direct eye contact during conversations [34]. Given the reliability and validity of the GARS, age- and gender-specific norms based on representative population samples would be useful to individualize diagnostic evaluations in the future.

## Conclusions

The German version of the GARS is a reliable and valid instrument with which to assess self-reported fear and avoidance of eye contact in various social situations. The two-factor structure of the original questionnaire has been replicated in the German version, indicating that fear and avoidance of eye contact can be differentiated in two categories of social situations: (1) everyday situations and (2) those involving high social threat. The GARS is well suited to assess the dispositional self-reported tendency to fear and avoid eye contact in social situations and might thus be useful as a sensitive measure for social anxiety in different clinical conditions such as SAD and other disorders associated with social impairments such as autism or schizophrenia.

## Supporting Information

S1 Appendix Augenkontakt Angst Skala. (PDF)
