# Supplementary material for: The German Version of the Gaze Anxiety Rating Scale (GARS): Reliability and Validity
Source: PLoS One. 2016 Mar 3;11(3):e0150807. doi: 10.1371/journal.pone.0150807 (PMC4777438; doi:10.1371/journal.pone.0150807)
Supplement: S1 Appendix — (PDF) [file pone.0150807.s001.pdf]

S1. Appendix

<u>Augenkontakt Angst Skala</u> (Gaze Anxiety Rating Scale – GARS)

Die folgenden Fragen beziehen sich darauf, ob Sie in unterschiedlichen Situationen Angst davor haben, Augenkontakt herzustellen, oder ob Sie Augenkontakt in diesen Situationen vermeiden. Wählen Sie Ihre Antworten in Erinnerung an die ***vergangene Woche*** – so, wie Sie sich in entsprechenden Situationen gefühlt und verhalten haben. Sollten Sie solche Situationen in der vergangenen Woche nicht erlebt haben, bewerten Sie bitte Ihre vermutete Angst vor Augenkontakt bzw. die Stärke des Drangs zu seiner Vermeidung.

Geben Sie nicht an, wie stark Ihre generelle Angst in der Situation war, sondern bewerten Sie nur den Grad Ihrer *Angst vor Augenkontakt* bzw. die *Stärke der Vermeidung*.

		**Angst**, Augenkontakt herzustellen<br><br>0 keine Angst<br>1 wenig Angst<br>2 mittelstarke Angst<br>3 starke Angst	**Vermeidung** von Augenkontakt<br><br>0 keine Vermeidung<br>1 wenig Vermeidung<br>2 mittelstarke Vermeidung<br>3 starke Vermeidung
1.	Eine Rede halten	0 1 2 3	0 1 2 3
2.	Mit einer Gruppe von Leuten auf einer Party reden	0 1 2 3	0 1 2 3
3.	Bei einer Besprechung das Wort ergreifen	0 1 2 3	0 1 2 3
4.	In einer Diskussion mit mehreren Leuten sprechen	0 1 2 3	0 1 2 3
5.	Beim Einkaufen mit einem Kassierer sprechen	0 1 2 3	0 1 2 3
6.	Jemandem vorgestellt werden	0 1 2 3	0 1 2 3
7.	Einen Bekannten im Vorbeigehen auf der Straße grüßen	0 1 2 3	0 1 2 3
8.	Mit jemanden sprechen, den Sie nicht gut kennen	0 1 2 3	0 1 2 3
9.	Mit jemanden sprechen, den Sie attraktiv finden	0 1 2 3	0 1 2 3
10.	Sich mit jemandem verabreden, den Sie nicht gut kennen	0 1 2 3	0 1 2 3
11.	In einer vertrauten Situation sein mit jemandem, der Ihnen nahe steht	0 1 2 3	0 1 2 3
12.	Mit Ihrem Chef oder Lehrer die Qualität Ihrer Arbeit diskutieren	0 1 2 3	0 1 2 3
13.	Eine alltägliche Unterhaltung mit einem nahen Familienmitglied führen	0 1 2 3	0 1 2 3
14.	Jemandem zuhören, der mit Ihnen spricht	0 1 2 3	0 1 2 3

		**Angst**, Augenkontakt herzustellen…<br><br>0 keine Angst<br>1 wenig Angst<br>2 mittelstarke Angst<br>3 starke Angst	**Vermeidung** von Augenkontakt<br><br>0 keine Vermeidung<br>1 wenig Vermeidung<br>2 mittelstarke Vermeidung<br>3 starke Vermeidung
15.	Mit jemandem sprechen, der Ihnen zuhört	0 1 2 3	0 1 2 3
16.	Eine Meinungsverschiedenheit zum Ausdruck bringen	0 1 2 3	0 1 2 3
17.	Ein Kompliment erhalten	0 1 2 3	0 1 2 3

Bitte beantworten Sie die folgenden zusätzlichen Fragen.

		0 überhaupt nicht<br>1 ein wenig<br>2 mittelstark<br>3 stark
18.	Ich vermeide Augenkontakt, weil er mir Angst macht.	0 1 2 3
19.	Ich vermeide Augenkontakt, weil er meine Konzentration stört (und nicht, weil er mir Angst macht).	0 1 2 3
20.	Ich fühle mich befangen, wenn ich Augenkontakt herstelle	0 1 2 3
21.	Ich befürchte, dass ich *zu lange* in die Augen meines Gegenübers starre.	0 1 2 3
22.	Es fällt mir schwer zu entscheiden, wie viel Augenkontakt am besten ist.	0 1 2 3
23.	Augenkontakt ist wichtig für meine privaten und beruflichen Beziehungen.	0 1 2 3

Wenn Sie keine Angst bzgl. des Augenkontaktes haben, dann kreuzen Sie hier an ______
und beantworten Sie die beiden folgenden Fragen nicht.

Beantworten Sie bitte noch die folgenden Fragen, wenn Sie Angst vor Augenkontakt haben oder Augenkontakt mit anderen Menschen vermeiden.

24. In welchem Alter hatten Sie zum ersten Mal Angst vor Augenkontakt bzw. haben Sie Augenkontakt vermieden? Alter:__________

25. Meine jetzige Angst vor, und die Vermeidung von Augenkontakt ist:

Schlimmer als in meiner Kindheit	0
Weder schlimmer noch besser als in meiner Kindheit	1
Etwas besser als in meiner Kindheit	2
Besser als in meiner Kindheit	3
Viel besser als in meiner Kindheit	4
